# Supplementary material for: Edible Seaweeds Extracts: Characterization and Functional Properties for Health Conditions
Source: Antioxidants (Basel). 2023 Mar 10;12(3):684. doi: 10.3390/antiox12030684 (PMC10045430; doi:10.3390/antiox12030684)
Supplement: Supplementary file 1 [file antioxidants-12-00684-s001.zip › antioxidants-2191992-supplementary.pdf]

Supplementary Materials:

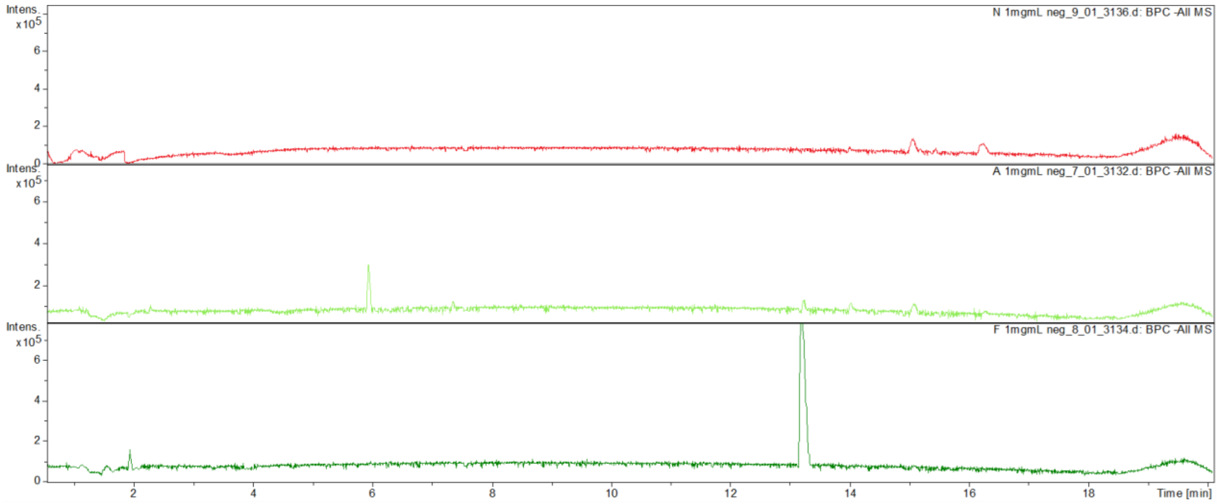

Figure S1 - Chromatographic profiles of the compounds in the three extracts, Nori (N), Aramé (A) and Fucus (F) obtained by using LC/HRMS-MS in ESI negative mode. Extracts at 1 mg dry mass/mL.
